# Ocular manifestations of patients with extranodal NK/T-cell lymphoma

**DOI:** 10.1038/s41433-025-04052-1

**Published:** 2025-10-07

**Authors:** Jia Luo, Rong Tao, Chuan-xu Liu, Yu-jie Ma, Victoria Y. Gu, Jing Li, Pei-quan Zhao, Ping Fei

**Affiliations:** 1 Department of Ophthalmology, Xinhua Hospital, Affiliated to Shanghai Jiao Tong University School of Medicine, Shanghai, China; 2 Department of Lymphoma, Fudan University Shanghai Cancer Center, Shanghai, China; 3 Department of Hematology, Xinhua Hospital, Shanghai Jiao Tong University School of Medicine, Shanghai, China; 4 Department of International Health, Johns Hopkins Bloomberg School of Public Health, Baltimore, MD USA

**Keywords:** Lymphoma, Uveal diseases, Retinal diseases

## Abstract

**Aims:**

To investigate the ocular symptoms and characteristics exhibited by extranodal NK/T-cell lymphoma (ENKTL) patients.

**Methods:**

One hundred sixty-eight patients with mature T/Natural Killer-cell Lymphoma (MTNKL) were recruited in this study, among whom 107 cases were ENKTL patients. A retrospective analysis was conducted on 12 of these ENKTL patients who had ocular symptoms. Results of ophthalmic and systemic auxiliary examinations, disease progression, and prognosis were documented.

**Results:**

Ocular symptoms occurred in bilateral eyes (*n* = 4) and unilateral eyes (*n* = 8). The average interval from the diagnosis of ENKTL to the onset of ocular symptoms was 18.1 (±20.8) months. According to the ocular symptoms and auxiliary examinations, eight patients had ocular adnexal symptoms only, and four patients had intraocular symptoms only. All patients exhibited impaired vision. After receiving combined treatment with systemic chemotherapy, radiotherapy, and local ocular therapy, six patients died during the follow-up, and the median survival time from diagnosis was 17 (range: 2–45) months. Among the surviving six patients, five out of seven eyes had poor visual prognoses lower than hand motion (HM), while two maintained a visual acuity of 20/25.

**Conclusion:**

In this study, the ocular presentations can be easily confounded with conditions such as uveitis or orbital cellulitis, necessitating vigilant clinical differentiation. To date, the visual prognoses of ENKTL patients with ocular symptoms remain regrettably poor.

## Background

Mature T-cell and NK-cell lymphoma (MTNKL) is a group of rare, heterogeneous, and high-mortality malignancies, comprising over 20 distinct disease entities according to the WHO classification system [1, 2]. Extranodal NK/T-cell lymphoma (ENKTL) is the most common subtype [3], which accounts for approximately 2.9% and 13.7% of the non-Hodgkin lymphoma cases in the United States and Northwest China, respectively [4, 5]. While the incidence of most ENKTL tends to rise with age and they frequently result in unfavourable clinical outcomes, survival rates have improved with new therapeutic approaches.

ENKTL can have devastating consequences for the ocular adnexa and eye. The nasal cavity, paranasal sinuses and nasopharynx are most commonly involved initially in ENKTL patients [6], while patients with orbital involvement are relatively rare [7]. Notably, most ocular manifestations are secondary, with primary intraocular lymphoma being less commonly reported [8, 9]. The low incidence of ocular involvement in ENKTL makes it difficult to comprehensively analyse its prevalence, progression, and prognosis. As such, our study aims to assess the characteristics and outcomes of patients with ENKTL who exhibit ocular manifestations.

## Methods

### Participants

Patients diagnosed with MTNKL between April 2019 and April 2022 at Xinhua Hospital were identified via the patient registry. The case inclusion criteria included: (1) a pathologically confirmed MTNKL diagnosis according to the WHO classification and (2) proven subtype classification. Diagnostic procedures included pathological biopsy and immunohistochemical staining. If required, in situ stains for Epstein–Barr virus (EBV)-encoded small RNA (EBER) and T-cell receptor gene rearrangement were detected using PCR. The diagnostic and classification criteria adhered to the latest WHO classification for tumours of hematopoietic and lymphoid tissues [1]. Positron emission tomography-computed tomography (PET-CT) and orbital magnetic resonance imaging (MRI) were used to monitor general and ocular metastasis. Comprehensive ophthalmic examinations for ocular manifestations included assessments of best-corrected visual acuity (BCVA), intraocular pressure, B-scan ultrasound, ultrasound biomicroscopy, Optos 200Tx and optical coherence tomography. The study was approved by the Ethics Committee of Xinhua Hospital, and informed consent was obtained from all participants.

### Treatment

The initial systemic therapeutic modalities for tumours varied widely, as a portion of the study cohort had received treatment at other institutions prior to referral. All patients received conventional chemotherapy, and early-stage patients with localised lesions received a combination of radiotherapy and chemotherapy. For patients presenting with ocular symptoms, treatment was tailored based on the specific local manifestations:

- Orbital infiltration cases: both systemic and local steroids were utilised for their anti-inflammatory effects. Patients with orbital mass received a systemic chemotherapy regimen, some also combined with radiotherapy, and when necessary, resection surgery was performed.
- Intraocular involvement cases: for manifestations like vitreous opacity, vitreous haemorrhage, or retinal detachment, interventions such as diagnostic vitrectomy and vitreous tap were recommended. If intraocular tumour metastasis was identified, intravitreal injections of methotrexate (400 μg/0.1 ml) were administered if accessible.

### Statistical analysis

Patients’ clinical data, including demographic information, clinical symptoms, ophthalmic findings, treatment modalities and outcomes, were recorded and summarised using descriptive statistics. Quantitative data were presented as median, and categorical data were expressed in terms of absolute and relative frequencies. All statistical analyses were conducted using SAS (version 9.4), and a *p* value < 0.05 was considered statistically significant.

## Results

### Patient characteristics

**Fig. 1:**
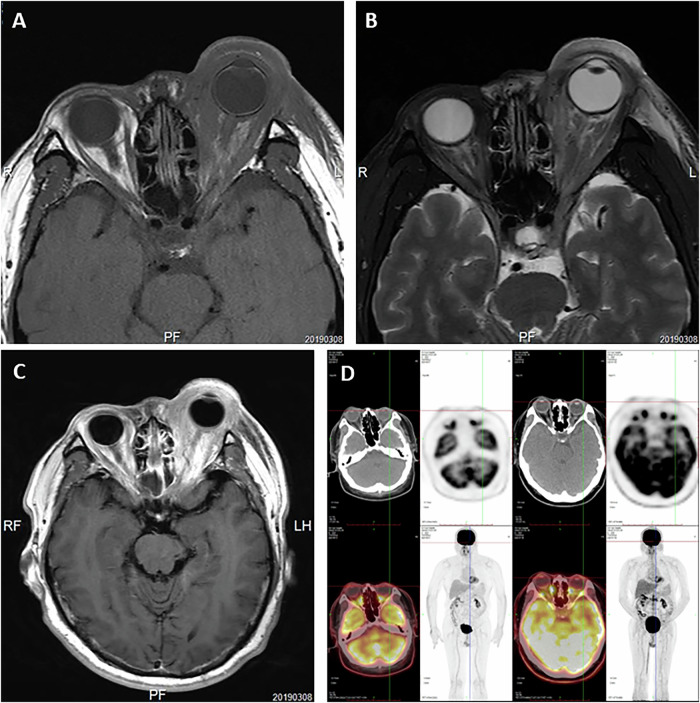
Ocular manifestations of ENKTL patients with ocular adnexal manifestations, case 3. **A**–**C** Orbital MRI: A poorly demarcated mass lesion is demonstrated in the left orbit involving the eyelids and periorbital tissues. The lesion exhibits an isointense signal on T1-weighted imaging and a hypointense signal on T2-weighted imaging, with marked contrast enhancement. The mass encases the globe with ill-defined margins between the lesion and both the optic nerve and extraocular muscles. **D** PET-CT: the left periorbital region shows patchy soft tissue density with mildly increased FDG uptake. The boundaries between the left extraocular muscles, optic nerve and surrounding soft tissues are indistinct. The orbital fat planes are obscured.

**Fig. 2:**
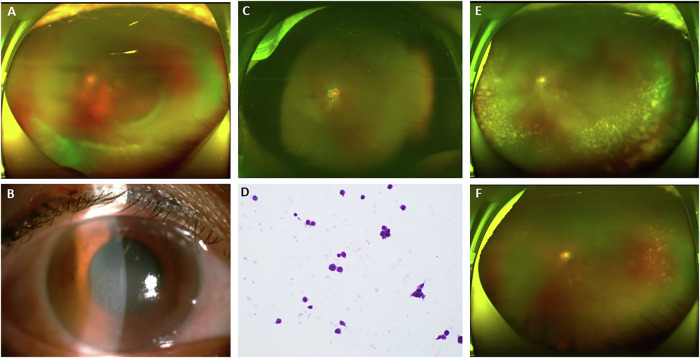
Ocular manifestations of ENKTL patients with intraocular manifestations, case 7. **A**, **B** Initial presentation: Ophthalmic examination revealed vitreous haze in the left eye, predominantly in the peripheral vitreous. The fundus showed a flat retina with a normal cup-to-disc ratio. Anterior segment photography demonstrated subtle corneal oedema and fine stellate keratic precipitates distributed diffusely over the inferior cornea. Tyndall (+), and no other remarkable abnormalities were noted. **C** Post-operation presentation: The vitreous cavity appeared clear following diagnostic pars plana vitrectomy, with flat retina and no detectable pathological changes. **D** Vitreous pathological analysis: Histopathological examination of the vitreous specimen by haematoxylin and eosin staining revealed scattered lymphoid cells without significant atypia, accompanied by occasional hyalocytes. The specimen contained sparse histiocytes with necrotic debris. **E** Disease progression: the patient failed to adhere to the recommended treatment regimen (intravitreal methotrexate injections), resulting in intraocular lymphoma recurrence characterised by recurrent vitreous opacities with multiple subretinal yellowish-white tumour nodules of varying sizes and shapes in the peripheral retina. **F** Therapeutic response: The intraocular metastatic lesions showed significant regression following multiple intravitreal methotrexate injections.

This study included a total of 168 patients with MTNKL, among whom 107 were ENKTL patients (Fig. S1). The median age of ENKTL patients was 46 (range: 11–84) years, and the male–female ratio was 1.82. The pathology for 14 patients with ocular symptoms was ENKTL (12 cases) and peripheral T-cell lymphoma, not otherwise specified (2 cases). Among these 12 ENKTL patients, 10 were male and 2 were female, resulting in a male-to-female ratio of 5. The median age for this group was 47 (range: 25–74) years. Demographic information and clinical characteristics such as age, gender, lymphoma subtype, initial tumour location, systemic involvement, treatment modalities and outcomes of ENKTL patients were recorded (Table 1). All ENKTL patients presented with nasal involvement, with 80% exhibiting the nasal cavity as the primary site, while one case manifested initial orbital symptoms. Among the 12 patients, 8 cases had unilateral eye involvement, with 3 cases affecting the right eye and 5 cases affecting the left eye; the remaining 4 patients had bilateral eye involvement. The average duration from ENKTL diagnosis to the onset of ocular symptoms was 18.1 (±20.8) months. Comprehensive systemic and ophthalmic examinations, including orbital or intraocular tissue biopsies, cranial and orbital MRI, PET/CT, and fundus examinations, classified patients into distinct categories, including ocular adnexal manifestations only (8, 66.7%) and intraocular manifestations only (4, 33.3%) (Figs. 1 and 2). Among the 16 eyes of the 12 patients studied, vision impairment was universally observed. Common signs for patients with orbital infiltration included orbital swelling, diplopia, ptosis, and either abduction or infraduction deficits. Patients with intraocular metastases exhibited clinical findings including keratic precipitates, vitreous opacity, vitreous haemorrhage, and subretinal yellowish-white tumour nodules. Detailed information is summarised in Table 2.

**Table 1:** Demographic information and clinical features of patients with ENKTL and ocular manifestations.

Patient number	Sex	Age (yrs)	Site of onset	EBV infection	Chemotherapy		Radiotherapy	Relapse	Follow-up time (mos)	Outcome
					Asparaginase-based regimens	PD-1 blockade-based regimens				
1	M	60	right nasal cavity	+	yes	yes	0	no	11	alive
2	M	42	bilateral nasal cavities	+	yes	yes	28/5040 cGy	yes	3	dead
3	M	37	left nasal cavity	+	yes	yes	1/not available	yes	25	alive
4	M	74	left maxillary sinus	+	yes	no	0	no	23	alive
5	M	63	bilateral nasal cavities	+	yes	yes	0	no	27	dead
6	M	63	right nasal cavity	+	yes	yes	0	yes	2	dead
7	M	49	left eye	+	yes	yes	0	yes	45	alive
8	M	45	right neck	+	yes	yes	0	yes	30	dead
9	M	55	left nasal cavity	+	yes	yes	0	yes	33	dead
10	F	25	bilateral nasal cavities	+	yes	yes	25/50 Gy	yes	33	alive
11	M	34	right nasal cavity	+	yes	yes	28/5040 cGy	yes	25	alive
12	F	34	bilateral nasal cavities	+	yes	yes	25/50 Gy	yes	25	dead

*M* male, *F* female, *yrs* years, *EBV* Epstein–Barr virus, *mos* months.

**Table 2:** Ophthalmic features and diagnostic frequencies of ENKTL patients with ocular involvement.

Ocular manifestations	Ocular adnexal only	Intraocular only	Total
Affected eye (%)	*N* = 11	*N* = 5	*N* = 16
Right eye	2	1	3
Left eye	3	2	5
Bilateral eyes	3	1	4
Average interval from diagnosis to ocular symptoms (mos)	10.6	33	18.1
Ocular symptoms (per patient)	*N* = 27	*N* = 17	*N* = 44
Abduction/Infraduction deficit	3	0	3
Diplopia	4	0	4
Ptosis	3	0	3
Orbital swelling	3	0	3
Cataract	0	1	1
Uveitis	0	3	3
Iridociliary mass	0	1	1
Intraocular hypertension	1	2	3
Macular oedema	0	1	1
Optic nerve infiltration	2	0	2
Retinal lesion	0	2	2
Retinal detachment	0	1	1
Vitreous haemorrhage	0	1	1
Vitritis	0	4	4
Vision decline/loss	10	2	12

*mos* months.

### Treatment and prognosis

All 12 patients received systemic chemotherapy treatment, and five patients received concurrent radiotherapy. Due to their refractory disease status, therapeutic strategies employed in this study consisted of asparaginase-based regimens and programmed cell death-1 (PD-1) inhibitor-based regimens with multiple chemotherapeutic agents (Table 1). Four patients did not receive topical treatment, among whom one patient had only mild ocular symptoms, and three patients had poor general health. One patient underwent orbital mass resection. Diagnostic vitrectomy and intraocular injection of methotrexate were performed in two patients with vitreous opacity and suspected intraocular tumour. Patients with ocular inflammation and high intraocular pressure were given symptomatic treatments. During a mean follow-up period of 23.5 (2–45) months, nine patients relapsed and six patients died of hemophagocytic syndrome or haemorrhagic shock. The mortality rate was as high as 50%. The mean survival time was 17 (2–45) months. For the seven eyes of the six surviving patients, only two eyes retained a BCVA of 20/25, while the rest (4 eyes) reported a BCVA lower than HM. The treatment and prognosis of ENKTL patients with ocular involvement were recorded in Table 3.

**Table 3:** Treatment frequencies and ophthalmic outcomes of ENKTL patients with ocular involvement.

Ocular manifestations	Ocular adnexal only	Intraocular only	Total
Systemic treatment			
Chemotherapy	8	4	12
Radiotherapy	5	0	5
Local treatment			
Anti-inflammation	4	4	8
Lower IOP	1	1	2
IVM	0	2	2
Retinal photocoagulation	0	1	1
Orbital mass resection	1	0	1
Visual acuity (per eye)			
NLP	3	1	4
LP-FC/BE	1	3	4
20/200-20/32	1	1	2
20/25-20/20	6	0	6

*IVM* intravitreal injection methotrexate, *IOP* intraocular pressure, *NLP* no light perception, *LP* light perception, *FC/BE* finger count/before eye.

## Discussion

In this study, we describe our experience in the diagnosis and treatment of ocular manifestations of ENKTL. We report an 8.3% incidence of ocular symptoms in 168 MTNKL patients, and the most common subtype was ENKTL (63.7%). The distribution was consistent with the dominant subtype of ENKTL in the Asian population in the literature [10]. For ENKTL patients, the median age for the 107 patients was 46 years, and was 47 years for the 12 patients with ocular symptoms. This is comparable to the median age of 41 years from a prior study [5], suggesting that ENKTL occurs predominantly in middle-aged adults. The male–female ratio of patients with ocular symptoms was 5:1, consistent with previous data showing an increased susceptibility to ocular manifestations in males [11].

Ocular manifestations in ENKTL predominantly involve the orbit as secondary lesions, mainly due to the direct invasion of adjacent structures in ENKTL [12], though orbital involvement may occasionally serve as the initial presentation [13, 14]. This study included one case that was presented with orbital swelling and visual impairment and had initially been misdiagnosed as cellulitis. This condition may delay correct diagnosis and treatment. Previous studies have reported two different types of ocular involvement in patients with nasal T/NK lymphoma, namely uveitis or vitritis and orbital infiltration [15]. Therefore, we support the recommendation that if the lymphoma does not respond to conventional therapy like steroids and antibiotics, timely use of orbital MRI and CT and biopsy is paramount [7].

ENKTL is a highly invasive and aggressive disease, with patients unfortunately facing poor prognoses and short survival times. ENKTL remains predominantly found in Asian and Latin American populations [16], intriguingly correlated to the geographic distribution of EBV subtype infections. In our study, all patients tested positive for EBV. The correlation between EBV infection and the progression of lymphoma is noteworthy and suggests varying stages of EBV ocular involvement, a hypothesis requiring further validation. Previous studies have shown that EBV-DNA copy number was associated with treatment response and disease prognosis in ENKTL [17, 18], and EBV-infected NK cells might further transform into malignant lymphoma [19]. Studies on the possible molecular pathogenesis of ENKTL have inferred that chronic active EBV infection may be the initial manifestation of EBV infection of NK cells, which progress to ENKTL when accompanied by additional genomic alterations from deficiencies in tumour suppressor genes and the constitutive activation of specific growth or transcription factors [20]. Given that EBV infection contributes to lymphoma progression, we posit that chronic active EBV infection, EBV-positive T-cell, NK-cell lymphoproliferative diseases, and NK/T-cell intraocular lymphoma metastasis may represent different stages of EBV involvement in the eye, for which further investigation is required.

Currently, combined chemotherapy and radiotherapy remain the most common therapeutic regimens for ENKTL patients [21, 22]. In most circumstances, timely systemic treatment is crucial, and local therapy is recommended as an adjunct, though efficacious treatments for ocular involvement are sparse [23]. Standard protocol calls for the surgical removal of tumours confined to the orbit. Diagnostic pars plana vitrectomies are generally recommended for cases of unexplained vitreous opacities or haemorrhage, and intravitreal methotrexate injection has been used for palliative and conservative vision-restoring measures [24]. All patients in this study received multi-agent chemotherapy regimens based on asparaginase or PD-1 inhibitors, and five of them received additional radiotherapy. Even though treatments had been administered accordingly, MTNKL patients with ocular manifestations exhibited significantly higher mortality (50%) compared to the overall MTNKL cohort mortality (27.4%) in this study, suggesting more aggressive disease biology in this subgroup. The median survival time from ENKTL diagnosis was 17 (range: 2–45) months, compared to the previously reported 21.7 (range: 2–69) months in patients with T-cell intraocular lymphoma [9], which aligns with the low survival rate observed in prior studies of ENKTL patients with concurrent ocular symptoms [14, 25, 26].

This study bears several limitations, including its retrospective nature, sample size for ENKTL with ocular involvement, and a homogenous population that may limit its generalisability, despite clinical features of the disease being generally similar [27]. Notably, MTNKL occurred less frequently than B-cell lymphomas in the cohort, resulting in a small sample size. Further, the mean follow-up time was 23.5 months, during which six patients died, making it difficult to investigate whether ocular involvement is an independent factor for the endpoint outcome of ENKTL patients. Lastly, tertiary referral bias may misrepresent the true incidence and nature of disease progression.

## Conclusions

In conclusion, ENKTL with ocular manifestation is a rare condition, but attention should be paid to differentiate it from other ocular diseases based on the prevalence of confounded diagnoses noted in this study. While general prognoses have improved due to aggressive regimens and novel therapies, the mortality rate of ENKTL and the vision-threatening nature of ocular involvement remain high and burdensome. We recommend future studies on the epidemiology and clinical outcomes for ENKTL patients with ocular symptoms and hope this study will enhance future discussions and collaborations with the lymphoma and ophthalmologic communities.

## Summary

### What was known before

- Extranodal NK-cell lymphoma is a rare and aggressive malignancy with a tendency to invade and involve adjacent tissues.
- Symptoms in most patients with extranodal NK-cell lymphoma are limited to the orbit.

### What this study adds

- Both the visual prognosis and the overall prognosis of extranodal NK-cell lymphoma patients with ocular symptoms were poor.
- With the increasing survival rates due to new therapeutic avenues, patients with extranodal NK-cell lymphoma need to be suspected for the presence of ocular involvement, including orbital infiltration and intraocular involvement.

## Supplementary information

Figure S1

## Data Availability

The datasets used and analysed during the current study are available from the corresponding author on reasonable request.
