# Supplementary material for: Ocular manifestations of patients with extranodal NK/T-cell lymphoma
Source: Eye (Lond). 2025 Oct 7;39(18):3243–8. doi: 10.1038/s41433-025-04052-1 (PMC12669774; doi:10.1038/s41433-025-04052-1)
Supplement: Supplementary file 1 — Figure S1 [file 41433_2025_4052_MOESM1_ESM.docx]

Fig.S1 MTNKL subtype distribution


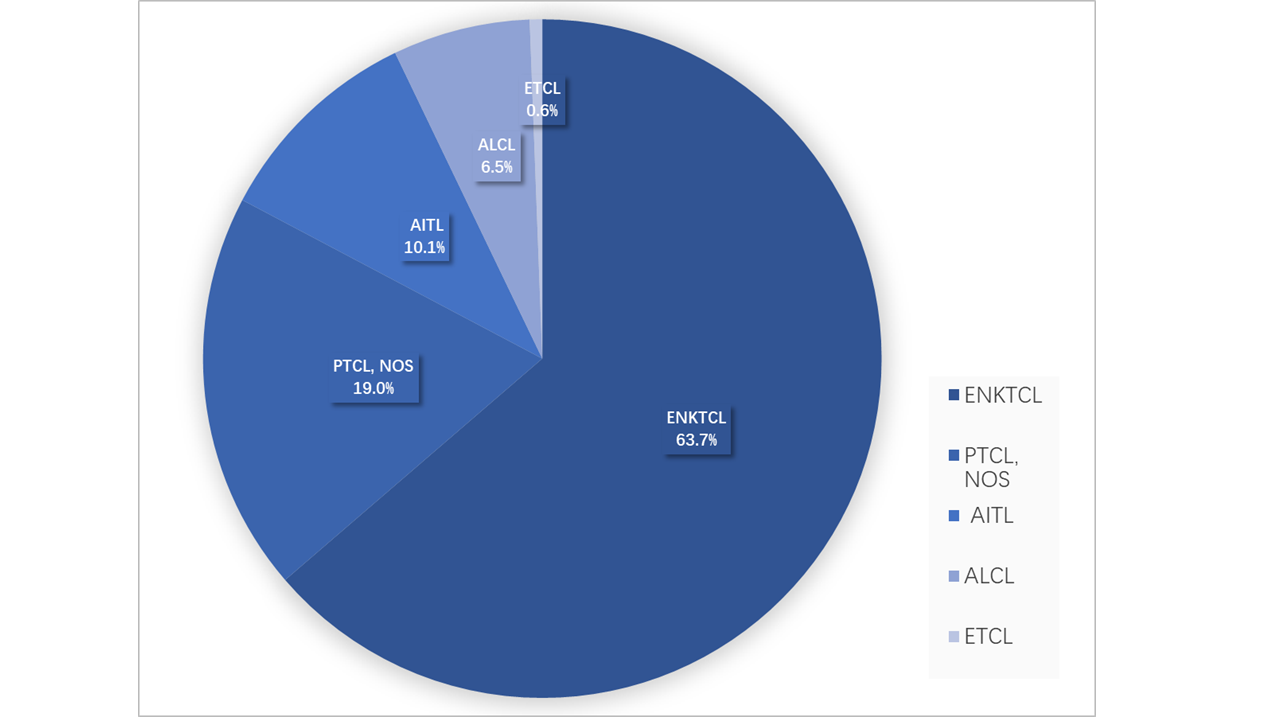


**Figure Legends**

Fig.S1 The pie chart shows the subtype distribution of total 168 MTNKL patients.
